# On the Treatment of Fever by the Application of Cold Water to the Surface of the Body

**Published:** 1847-01

**Authors:** J. H. Stallard

**Affiliations:** Surgeon to the Leicester General Dispensary


### III. ON THE TREATMENT OF FEVER BY THE APPLICATION OF COLD WATER TO THE SURFACE OF THE BODY.

BY J. H. STALLARD, ESQ.,
Surgeon to the Leicester General Dispensary.

*(In a Letter to John Forbes, M.D., F.R.S.)*

[We have great pleasure in laying the following communication before our readers, as the first fruits of our attempt to rouse anew the attention of the legitimate members of the profession to the great value of cold water as a therapeutic agent, and thus, if possible, to stimulate them to rescue its use from the hands of ignorant non-medical pretenders and charlatans. Mr. Stallard, like a man of sense, did not hesitate to apply what he thought would benefit his patients, because the mode of treatment might have first been used by one ignorant of all science, or since practised by some devoid of all honesty. He adopted the good as he found it; but, like a scientific practitioner, applied the remedy according to the rules of science,—at the proper time, in the proper cases, and in the proper manner. It is thus we would have the members of the profession to act generally, and so bring under the domain of scientific medicine, and within the pale of professional practice, what must now be considered as a weapon of power in the hands of our enemies;—as we may truly call those who profess to use the hydropathic or any other method of healing diseases, while ignorant of the knowledge on which alone rational practice can be based. To say that cold water employed in the mode lately introduced by Priessnitz is not a powerful therapeutic agent, is simply to express ignorance of a common and well-known fact; and to admit its potency as an agent, is equivalent to saying that, improperly administered, it is capable of doing mischief. In fact, we know that it is capable of doing great mischief, and believe that it has done great mischief; but it is also capable of doing great good; and we have called upon the members of the medical profession to destroy the evil, and cherish and promote the good, by taking the practice—or as much of it as is warranted by fair evidence—into their own hands, and modifying it in accordance with the laws of science, and in obedience to rational experience.

We are far from asking our readers to adopt hydropathy in an exclusive form, much less to become professed hydropathists; on the contrary, we ask them to assist us in destroying hydropathy as it is now practised, and in routing the whole phalanx of trading water-doctors, by cutting from under them the ground on which rests their strongest hold on public patronage—viz. the pertinacious and scornful rejection by the profession of certain modes of treatment employed by them, which the public have found to be useful, and choose to patronize accordingly.

In making the attempt to awaken attention to this subject, at the time and in the manner we made it, we were well aware that we should excite opposition not merely from the ignorant and self-interested, but even from some who were qualified to judge of the question, both as a matter of science and as a matter of professional propriety, but who had not had any experience of the facts, or well considered the subject in a scientific point of view. But here, as in the previous discussions in the Journal, on Homœopathy, Mesmerism, and Phrenology, it required no effort on our part to meet any opposition that might be excited, or to tolerate with perfect calmness any criticisms that might be addressed to us: the importance of the object to be attained—the singleness of our aim—the consciousness of proper motives—were sufficient to render us perfectly satisfied with the result, whatever it might be, and to keep us steadily and quietly advancing in the humble path which we had deliberately chosen. Our first object in what we have done, and are doing, is the attainment of truth; our next is the maintenance and promotion—if it may be—of the honour, and dignity, and true interests of the medical profession. In the attainment of these objects, or in the endeavours to attain them, we trust we shall never so far forget ourselves as to prefer, at any time, the wrong because it is fashionable or safe, or to abandon the right because its advocacy may expose us to some inconvenience, to the imputation of unworthy motives, or even to the charge of a dereliction of professional duty. He is a poor politician and a poorer philanthropist who thinks only of the present, and cares only for the pleasing. We would fain hope that we of the British and Foreign are made of sterner stuff.]

Leicester, Nov. 18, 1846.

MY DEAR SIR,—Although personally unknown to you, I have ventured to send you the inclosed paper, containing notes of some cases of fever treated by me in the Leicester Temporary Fever House by the application of cold water. During the autumn, fever has raged here to a degree far greater than usual, and the board of guardians have established a temporary fever house, in which I acted as resident surgeon for the space of one month. It was during this residence that the last number of your Journal, containing your article on hydropathy, and remarks on the treatment of fever pursued by Dr. Currie, came under my notice. This so engaged my attention that I requested my father, one of the union medical officers, to place his share of cases under my charge, in order that I might give cold water a fair trial. The cases I have reported are not *selected* cases; they are those which presented themselves as being most proper for the trial; and are all which came under my *entire* observation in the fever institution. I may, however, remark that the same plan has since been carried out by my father in all recent cases with the most decided success, and I myself have frequently employed it with like benefit in private practice. The mode of proceeding was as follows:

The patient was stripped naked, enveloped in a *cold* wet sheet, and covered with a blanket. After remaining in this situation from ten to fifteen minutes he was, without being dried, immediately wrapped in a blanket thoroughly heated before the fire, and thus removed to another bed and covered over with bedclothes. The effect produced by the wet sheet is, first, a sensation of great cold, accompanied with sighing; but this is almost immediately succeeded by an agreeable sensation of coolness and comfort; the sheet then begins to grow warm, and when the heat of the skin had been previously very great, the blanket reeks with steam. Shortly after the patient is removed to the warm bed he begins to perspire, his headache and muscular pains cease, and he sinks into a calm and undisturbed sleep, from which he awakes still perspiring, but painless, refreshed, and occasionally well.

With respect to the character of the fever which has so extensively prevailed in this neighbourhood, I believe it to be the common typhoid fever of this country. Its type has varied considerably; for the most part its tendency has been enteric; but during a few weeks, and that especially during the observations I have detailed, there was a marked tendency to pneumonic implication, chiefly of an asthenic character. With a few exceptions, I have not observed much cerebral disease. The following extracts will show the general features of the disorder:

LEICESTER UNION FEVER INSTITUTION.

| | |
|---|---|
| Number of patients admitted from Sept. 12th to Nov. 10th | 187 |
| Remaining at time of the Report | 30 |
| Discharged | 157 |
| Of whom died | 16 |
| Cured | 141 |

Average deaths, 10·1 per cent.

Time the patients remained in the institution:

| | |
|---|---|
| Longest | 50 days |
| Shortest (case of Smith) | 2 ,, |
| Average | 12 ,, |

Time of residence of the fatal cases:

| | |
|---|---|
| Longest | 50 days |
| Shortest | 1 day |
| Average | 12 days |

LEICESTER COUNTY FEVER INSTITUTION.

| | |
|---|---|
| Patients admitted from Aug. 1st, to Oct. 31st | 99 |
| Remaining at the time of report | 14 |
| Discharged | 85 |
| Cured | 70 |
| Died | 15 |

Average deaths, 15·2 per cent.

Time the patients were resident in the institution:

| | |
|---|---|
| Longest | 83 days |
| Shortest | 4 ,, |
| Mean | 25 ,, |

Time in the institution of the fatal cases:

| | |
|---|---|
| Longest | 39 days |
| Shortest | 4 ,, |
| Average | 16 ,, |

Duration of illness in fatal cases prior to admission.

| | |
|---|---|
| Longest | 28 days |
| Shortest | 1 ,, |
| Mean | 11 ,, |

It will be seen by these tables that the deaths in both institutions resulted principally from the admission of hopeless cases. The form of complaint experienced in the County Fever House was more severe, and longer in its duration, than in the sister institution—no doubt arising from the neglect which country people more frequently suffer before admission; whereas the Union Fever House being placed under the care of their own medical officers, the latter were enabled to remove every case as it appeared; thus very materially diminishing the average residence of the patients, and also the per centage of deaths. On the whole, the mortality of the town has considerably exceeded the usual average. The deaths, as far as my own examinations tell, have resulted either from ulcerations of the small intestines, or congestive or typhoid pneumonia.

REMARKS ON THE CASES.

In the first case, the patient had a slight cough before the cold application, and it appeared as though the cold had rather increased the internal congestion; but the general state of the boy clearly showed that the constitution was entirely relieved by the profuse perspiration. The cough did not return with the second application of cold.

Case II was one in which I hesitated to use the cold, fearing lest the internal organs, which were already seriously affected, should suffer by it; but the disease seemed so really serious, the skin was so remarkably harsh and dry, no perspiration having ever taken place since his admission, that I determined to try it. The effect was most gratifying; the perspiration seemed at once to unload the system, to restore the functions of the lungs and alimentary canal to something like order, and the stimulants ordered at the same time aided, I have no doubt, in bringing about this desirable and, to me, unexpected result.

The third case was equally successful. Although the patient had been under the ordinary treatment five days without real benefit, he was at once relieved by the cold sheet. The application in this case, as in most others, caused *immediate costiveness.* This is remarkable; for the general type of the epidemic has been characterized by the frequent occurrence of diarrhea.

Of the following cases I shall only notice the eighth and tenth; the former being by far the most striking cure resulting from the cold-water treatment—a single application, without any medicine, having restored the healthy condition of the body.

The last case, although terminating fatally, does not furnish a single argument against the use of the cold sheet. The death was, no doubt, caused by sudden congestion of the lungs, which fever patients are especially prone to, and the tendency to which was probably augmented by the dose of morphine administered over night to allay the excessive restlessness. The application of the cold itself was followed by the best result, viz. free perspiration; which I am afraid was checked by the restlessness of the patient causing the bedclothes to be thrown off and the body to be exposed. I have related it with the rest; and, although I should, perhaps, hesitate again to employ the sheet in the very advanced stage of fever, especially where there is great irritability of the nervous system, I think its use must be highly advantageous in debilitated persons when first seized, as it enables the practitioner to employ beef-tea, wine, and other stimulants, whilst, at the same time, means are being used to restore the action of the skin and the healthy condition of the blood.

I shall not tire your patience with remarks on the pathology of fever, or the theory of its *cure;* but, before concluding, I shall offer one or two observations which have occurred to me during the epidemic of the present autumn. At its commencement I very frequently employed an emetic at the first onset of the febrile attack, and with very varied success. In many cases, free vomiting was accompanied by profuse perspiration, in a few by purging also; and these cases, without exception, got well immediately; but in other instances, and those not a few, no perspiration ensued, and in these as constantly did the tongue become dry and foul, the thirst and fever increase. I think that this observation, together with the evidence afforded by the cases I have recorded, clearly shows that, in this epidemic at least, a fever may be cured, and that not by a rash, uncertain, and empirical mode, but by a remedy which coincides with and favours the efforts of Nature. This I think the cold sheet, as applied by me, does, first, by relieving the system of caloric which it is endeavouring to get rid of by over-action of the skin and lungs; secondly, by reducing the temperature of the surface, and so enabling the skin to resume its healthy excretory action; and, thirdly (through the natural stimulus given to that healthy action), by depurating the blood of those effete matters which the checked excretion of the skin had locked up in it.

Should you think the inclosed cases likely to be of any public use, I shall feel a pleasure in having recorded them; remembering, as I do, how deeply I am indebted to you for the instruction and benefit I have always received from the perusal of your valuable Journal.

I remain, dear sir, yours, most truly,

J. H. Stallard.

---

*Notes of Cases of Fever treated in the Leicester Temporary Fever Institution, October* 1846.

Case I. Coleman, Henry, æt. 10, admitted October 5th, from a close and most unhealthy yard. He complained of rigors; skin very hot and dry; bowels quite open; slight cough. He was ordered simple febrifuge mixture, with low, simple diet.

October 6th. The skin very hot and dry; the tongue red and very foul; bowels open; he complains of slight cough and headache. There is no apparent congestion of the lungs.

He was ordered to be surrounded with a cold wet sheet, to be placed in a blanket, and to remain in it for ten minutes.

*Vespere.* About half an hour after the cold application he broke out in a profuse perspiration, which continued during the whole day. He appears much better this evening, is more lively, and his head is relieved; still slight cough. Contin. med.

7th. Tongue cleaner, moist; bowels open; complains of headache. His nose has bled during the night. Cough as before. Slight crepitation observed at the base of the left lung, but he has perspired freely through the whole night.

To have cold water applied to the head, and to continue the fever mixture.

8th. Much better; crepitation gone; no recurrence of fever. Ordered to get up.

9th. There was a slight recurrence of fever last night, and he was again placed in the wet sheet; perspiration was induced, and it continued during the whole night. His tongue clean, and bowels regular; cough gone. To have rice pudding, and omit medicine.

10th. Continues improving. To have beef-tea. 11th. Discharged cured.

Case II. Tomlinson, Spring, æt. 26, admitted October 2d, consequently had been ill a week when I first saw him on Oct. 9th. He had been taking simple fever mixture, and had had diarrhœa and pneumonia. His tongue was dry and foul; bowels still purged; cough; mucous râles were heard over both sides of the chest, at the back part; pulse very feeble, upwards of 100; skin had never been moist since his admission, and is now remarkably harsh and dry. Ordered the wet sheet to be applied as above, and to have 1½ oz. of port wine every four hours, with a little beef-tea. No medicine.

10th. Much relieved. Shortly after the removal of the wet sheet he broke out into a most profuse perspiration, which has not yet entirely gone off. Tongue is now clean and moist; his bowels have been open twice. He had no beef-tea yesterday, from an error of the nurse, but it was again ordered.

11th. Skin is quite wet; his cough is gone; his tongue is much improved; and his bowels have been costive since yesterday's visit. To have ol. ricini. ℥ss.

12th. Better. Bowels opened by the oil; he coughs occasionally; but a thick whitish phlegm is easily expectorated. To have meat diet [too soon ?]

13th. Tongue dry; bowels open twice; no thirst; his skin is quite moist; and otherwise he is much better. To omit meat, and recur to beef-tea.

14th. Tongue is to-day clean and moist.

He continued to improve, and was discharged on the 16th.

Case III. Thomas Harris, æt. 14, admitted October 3d. He had been ill seven days previous to his admission, but had so far improved that he was ordered to get up on the 7th. On the 8th I found him with a foul tongue, very red at the tip and margin; bowels open once in twelve hours; great thirst; aching of limbs, back, and head; a frequent pulse; and his skin very hot and dry. He was ordered the wet sheet and no medicine.

9th. He perspired freely after the cold application, and the perspiration continued all night, but had entirely ceased at the time of my visit, 9 a.m. The tongue is still very foul and red. To have the wet sheet again this evening.

10th. Skin is now quite wet with perspiration, which has been the case ever since the cold application; the tongue is not improved; the bowels are costive; the pulse is much more feeble; and he complains of weakness, but neither of fever nor headache. To have ol. ricini ℥ss; to be repeated in four hours if necessary. The cold application to be repeated if the fever should return.

11th. Better. Bowels open; tongue cleaner. The skin preserved its moisture all day yesterday, so that the wet sheet was not employed; but as the skin is now hotter and drier than at my visit yesterday, it was ordered to be used to-day. The pulse is more feeble, and he was ordered half a pint of beef-tea in the twenty-four hours.

12th. Very much improved. The tongue is clean; the bowels are open; perspiration occurred after the cold sheet, and has continued ever since; the skin is now quite cool; he complains of weakness.

13th. Better; and he was ordered meat diet.

The convalescence was at once established, and he was discharged on the 15th.

Case IV. Joseph Timson, æt. 23, married, admitted October 8th. He had been seized with shivering on the 4th, and had had an attack every day since. He complained of muscular prostration, pain of head, limbs, and back, great thirst, and loss of appetite; his tongue was foul; his skin hot and dry; and his bowels costive. He was ordered the wet sheet immediately.

9th. Almost well. He perspired freely after the application of the cold sheet, and the perspiration still continues. His pain of head and limbs is gone; the tongue is foul, and his bowels are costive, which makes him feel uneasy. To have ol. ricini ℥j, and wet sheet if necessary.

10th. Perspiration has continued. The skin is quite cool and moist; the tongue is much cleaner; and the bowels are quite open. He was ordered meat diet, and to get up.

11th. Skin still continues moist. He feels much better; his tongue is not quite so clean; and his bowels have not been moved since yesterday. Repeat ol. ricini.

12th. Same as yesterday. Bowels still costive. Repeat ol. ricini.

13th. Feels quite well; his appetite is good; he has no return of shivering or fever; his bowels are freely open; and he wishes to go home. Discharged.

Case V. John Foreman, æt. 16, admitted October 5th; but the wet sheet was not applied until the 8th. His state was little better than when he was admitted; his bowels were purged; his skin was very hot and dry; and his tongue very foul.

9th. He is greatly relieved, having perspired freely ever since the application. He was ordered to have it repeated if the fever should return.

10th. Much the same as yesterday. The sheet was not again applied.

11th. Much the same. There was a return of fever last night, and his skin is not very moist, and rather hot. The wet sheet was administered.

12th. Has perspired freely all night; his tongue is clean; bowels open. Ordered to get up; to have rice pudding.

13th. Says he feels quite well. His discharge was delayed until the 15th.

Case VI. Foreman, Mary Ann, sister of the above, æt. 20, single, was attacked with shivering fourteen days back, but continued her work until the 14th of October. She was admitted on the 16th, late in the evening.

17th. Had a shivering fit after her admission last night, and when reaction came on she had the cold sheet applied as in the former cases. I found she had perspired freely all night, but her skin, though still moist, was very hot; her tongue was foul and red; her bowels open. She complained of great prostration and headache. She was ordered the liq. ammon. acet. and camphor mixture every four hours, low diet, and the wet sheet at night.

18th. She has been in a comfortable perspiration all night. The tongue is much improved; bowels are costive; headache much relieved. Cont. med.; omit the sheet.

19th. Bowels still costive, but tongue nevertheless cleaner. She has perspired freely, and her skin is now cool and moist. Omit medicine. To take ol. ricini ℥ss.

20th. Convalescent. Her discharge was delayed two days, on account of her peculiar work.

Case VII. Thomas Kean, æt. 19, admitted October 9th, from a dirty yard, where fever has been very severe. Had been seized with shivering on the 7th, and when seen was in a high state of fever, and was specially noted as promising to be a very bad case. I sent him immediately to the fever house, with orders to place him in the wet sheet immediately. This was done for ten minutes, and about twenty minutes after the sheet was removed he began to perspire.

10th. Relief is very marked. He has had a good night, and his head and back aches are gone, whilst his skin continues quite wet with perspiration; his bowels are costive. To have low diet, and ol. ricini ℥ss.

11th. His skin became hotter last night, and his pain and thirst slightly returned, and the wet sheet was therefore repeated. He is not much better. His tongue is clean, though red; bowels have been relieved four times. He was to get up, and have rice pudding.

12th. Bowels costive. Complained of headache towards the afternoon. Ordered to have the sheet if necessary, and ol. ricini ℥ss directly.

13th. Tongue less red; bowels open. Did not require the sheet; headache relieved.

14th. Better. He was discharged cured the day following.

Case VIII. Jane Smith, æt. 42, mother of six children, was admitted October 11th. She had been seized with shivering fourteen days before her admission, and had felt chills ever since. She was taken much worse on the 9th, so as to be unable to attend to her family. Her bowels were very costive, and on the morning of the 9th she took salts and senna, which have acted very violently. She now complains of intense headache, and aching of the back and limbs, with thirst and great prostration. She was ordered the wet sheet and low diet; no medicine.

12th. Perspiration came on after the cold application, and has continued all night most profusely. The headache and lumbar pain are quite gone, and she complains only of slight weakness. To have meat diet, but to remain in the fever house.

13th. No return of shivering or fever. Her skin is moist, and tongue clean; feels very feeble, but is anxious to go out, as she thinks she can attend to her family. Discharged.

Case IX. T. Cooper, æt. 30, living in a dirty, ill-ventilated house, was seized with shivering on the 9th of October, and has been unable to do anything since, the shivering having occurred every day, accompanied with the usual symptoms of the epidemic. When admitted on the 12th, he complained more especially of thirst, sore throat, and back-ache. His tongue was foul; his skin hot and dry; and his bowels reported open. Ordered to have the wet sheet.

13th. Has been in a good perspiration all night. Tongue is much cleaner; his bowels are costive. Altogether he feels much better, but he still complains of his throat. The fauces were red and swollen. Ordered ol. ricini ʒvj, and a mustard sinapism to the throat.

14th. Bowels not well relieved; skin cool and moist; tongue cleaner. To repeat the ol. ricini.

15th. Much better. Bowels freely open; no headache. To have meat diet.

18th. Discharged cured.

Case X. Elizabeth Holmes, æt. 12, had been confined to bed a fortnight, admitted on the evening of October 16th, and first seen on the morning of the 17th. She had had a very restless night, and had moaned frequently. She was quite sensible, and complained of no pain. Her tongue is dry, brown, and cracked; the skin hot, very harsh, and dry. She has had frequent sickness, which is always removed by the exhibition of the medicine which she had been taking under the direction of her previous medical attendant. Bowels have been moved three times since her admission; there is no pain or tympanitis; pulse very feeble; the second stroke of the heart very indistinct; no cough or congestion of the lungs. To take potass. bicarb. gr. vj, ex aquâ 4tis horis; to have wine and beef-tea, and to have the wet sheet in the evening.

18th. She perspired after the cold application, and the perspiration continued until 1 a.m. to-day; after this she was very restless, and crying. Bowels are open; tongue same as yesterday; sickness ceased after the exhibition of the medicine; urine moderate in quantity high-coloured, clear; Cont. Mist. To have morphiæ acet. gr.$\frac{1}{8}$ horas omni. Diet as before. The sheet not to be repeated.

19th. She slept a little after the draught, but again became very restless, About 1 a.m. dyspnœa came on, which increased until 11 a.m., when she died. I saw her about 8 a.m., when the powers were failing, and the lungs were much congested. No post-mortem examination was allowed.

